# Quality of life and associated factors in institutionalized older adults: implications for gerontological nursing. Analytical study

**DOI:** 10.15649/cuidarte.5800

**Published:** 2025-12-19

**Authors:** Claudia Consuelo Torres Contreras, Albeiro Vargas Romero

**Affiliations:** 1 Ministerio de Ciencia, Tecnología e Innovación, Bucaramanga, Colombia. E-mail: claudiaconsuelo@yahoo.com Ministerio de Ciencia, Tecnología e Innovación Bucaramanga Colombia claudiaconsuelo@yahoo.com; 2 Fundación Albeiro Vargas Y Ángeles Custodios, Bucaramanga, Colombia. E-mail: albeirovargas@yahoo.com Fundación Albeiro Vargas Y Ángeles Custodios Bucaramanga Colombia albeirovargas@yahoo.com

**Keywords:** Quality of Life, Aged, Institutionalization, Geriatric Nursing, Physical Activity, Calidad de Vida, Persona Mayor, Institucionalización, Enfermería Geriátrica, Actividad Física, Qualidade de Vida, Idoso, Institucionalização, Enfermagem Geriátrica, Atividade Física

## Abstract

**Introduction:**

Population aging and the rise in chronic diseases demand strengthening strategies to promote the well-being of institutionalized older adults. The FUMAT scale enables the assessment of quality of life across eight dimensions of well-being.

**Objective:**

To analyze the factors associated with the quality-of-life profile of institutionalized older adults.

**Materials and Methods:**

A cross-sectional analytical study was conducted with 250 permanent residents (≥3 months) of a nursing home in Bucaramanga, Colombia. Individuals aged 60 years or older with a reliable informant were included; those with terminal illness, acute psychiatric disorders, or clinical instability were excluded. The FUMAT scale, Barthel Index, PULSES profile, FRAIL scale, Downton Fall Risk Index, and the Short Portable Mental Status Questionnaire (SPMSQ) were applied. Descriptive and bivariate analyses were performed using Pearson's chi-square test, Fisher’s exact test, and the Mann–Whitney U test. Multivariate analysis was conducted using binary logistic regression. Data were processed using Stata v17.

**Results:**

The lowest-scoring dimensions were material well-being and interpersonal relationships, whereas rights and social inclusion achieved the highest scores. Physical activity and participation in productive activities were associated with better scores on the FUMAT Quality-of-Life Index (FUMAT-QOLI). In the multivariate model, cognitive impairment (OR=0.34; 95% CI: 0.12–0.91) and motor impairment (OR=0.30; 95% CI: 0.14–0.64) significantly reduced the likelihood of belonging to the highest FUMAT-QOLI tertile; physical and productive activities showed a positive but non-significant trend.

**Discussion:**

The findings indicate that functional capacity and cognitive integrity are key determinants of well-being among institutionalized older adults. Low scores in material well-being and interpersonal relations suggest resource and social support limitations, requiring institutional intervention. The association between activity and well-being supports the implementation of active aging programs.

**Conclusion:**

Promoting autonomy, cognitive stimulation, and social integration is essential to improve quality of life.

## Resumen

**Introducción:**

El envejecimiento y el aumento de enfermedades crónicas exigen fortalecer estrategias que promuevan el bienestar de las personas mayores institucionalizadas. La escala FUMAT permite evaluar la calidad de vida a partir de ocho dimensiones del bienestar.

**Objetivo:**

Analizar los factores asociados al perfil de calidad de vida en personas mayores institucionalizadas.

**Materiales y métodos:**

Estudio transversal analítico con 250 residentes permanentes (≥3 meses) de un centro gerontológico en Bucaramanga, Colombia. Se incluyeron personas de 60 años o más con informante confiable; se excluyeron quienes presentaron enfermedad terminal, trastornos psiquiátricos agudos o inestabilidad clínica. Se aplicaron la escala FUMAT, el índice de Barthel, el PULSES profile, la escala FRAIL, el índice de riesgo de caída Downton y el Short Portable Mental Status Questionnaire (SPMSQ). Se realizaron análisis descriptivos y bivariados mediante las pruebas chi-cuadrado de Pearson, exacta de Fisher y U de Mann- Whitney. Para el análisis multivariado se utilizó regresión logística binaria. Los datos fueron procesados en Stata v17.

**Resultados:**

Las dimensiones con menores puntajes fueron bienestar material y relaciones interpersonales, mientras que derechos e inclusión social obtuvieron los valores más altos. La actividad física y la participación productiva se asociaron con mejores puntajes del Índice de Calidad de Vida FUMAT (ICV-FUMAT). En el modelo multivariado, la discapacidad cognitiva (OR=0.34; IC95%: 0.12–0.91) y la discapacidad motora (OR=0.30; IC95%: 0.14–0.64) redujeron significativamente la probabilidad de pertenecer al tercil superior del índice; la actividad física y productiva mostró tendencia positiva sin significancia.

**Discusión:**

Los hallazgos evidencian que la funcionalidad y la integridad cognitiva son determinantes del bienestar en personas mayores institucionalizadas. Las bajas puntuaciones en bienestar material y relaciones sugieren carencias en recursos y apoyo social que requieren intervención institucional. La relación entre actividad y bienestar respalda programas de envejecimiento activo.

**Conclusiones:**

Fomentar autonomía, estimulación cognitiva e integración social es esencial para mejorar la calidad de vida.

## Resumo

**Introdução:**

O envelhecimento populacional e o aumento das doenças crônicas exigem estratégias que promovam o bem-estar de idosos institucionalizados. A Escala FUMAT permite avaliar de forma abrangente a qualidade de vida a partir de oito dimensões do bem-estar.

**Objetivo:**

Analisar os fatores associados ao perfil de qualidade de vida de idosos institucionalizados.

**Materiais e métodos:**

Estudo transversal analítico realizado com 250 residentes permanentes (≥3 meses) de um centro gerontológico em Bucaramanga, Colômbia. Foram incluídos indivíduos com 60 anos ou mais e com informante confiável; excluíram-se aqueles com doença terminal, transtornos psiquiátricos agudos ou instabilidade clínica. Foram aplicados os instrumentos: Escala FUMAT, Índice de Barthel, PULSES profile, escala FRAIL, Índice de Downton e Short Portable Mental Status Questionnaire (SPMSQ). Realizaram-se análises descritivas e bivariadas utilizando os testes Qui-quadrado, Fisher e Mann–Whitney. A análise multivariada foi conduzida por regressão logística binária. Os dados foram processados no Stata v17.

**Resultados:**

As dimensões com menores escores foram bem-estar material e relações interpessoais, enquanto direitos e inclusão social apresentaram os valores mais altos. A prática de atividade física e a participação em atividades produtivas associaram-se a melhores escores do Índice de Qualidade de Vida FUMAT (IQV-FUMAT). No modelo multivariado, a deficiência cognitiva (OR=0.34; IC95%: 0.12–0.91) e a deficiência motora (OR=0.30; IC95%: 0.14–0.64) reduziram significativamente a probabilidade de pertencer ao tercil superior do IQV-FUMAT; atividades físicas e produtivas mostraram tendência positiva, porém sem significância estatística.

**Discussão:**

Os achados indicam que a capacidade funcional e a integridade cognitiva são determinantes centrais do bem-estar de idosos institucionalizados. Baixos escores em bem-estar material e relações interpessoais revelam limitações de recursos e apoio social que requerem intervenção institucional. A associação entre atividade e bem-estar reforça o valor dos programas de envelhecimento ativo.

**Conclusões:**

Promover autonomia, estimulação cognitiva e integração social é essencial para melhorar a qualidade de vida.

## Introduction

Increased life expectancy and declining birth rates have led to an inversion of the population pyramid^1^. In Colombia, the National Administrative Department of Statistics (DANE) reported a 14.6% decline in births^2^, while projections from the World Health Organization (WHO) indicate sustained growth in population ageing both globally and across Latin American countries^3^. This phenomenon requires that care for older adults focus not only on prolonging life, but also on ensuring optimal living conditions. The increase in chronic diseases and the growing demand for specialized services have reinforced the role of nursing as a fundamental axis in promoting active and dignified aging^4^. From this perspective, the study and implementation of strategies aimed at improving quality of life become a priority, considering dimensions such as physical and emotional well-being, interpersonal relationships, self-determination, personal development, and social inclusion^5^,^6^.

Globally, the inversion of the population pyramid is progressing rapidly, particularly in developing countries, resulting in aging communities^7^. This process, together with age-related physiological changes, increases the risk of falls, functional decline, and increased mortality, thereby affecting quality of life, particularly among institutionalized individuals. Understanding these factors is essential for nursing to design interventions that promote autonomy, social participation, and functional and cognitive integrity^8^–^10^.

The quality of life in later life is a multidimensional concept influenced by socioeconomic factors, lifestyles, health, housing, and social support^11^. In Colombia, the population is undergoing rapid aging: the percentage of older adults rose from 6.9% in 1985 to 13.8% in 2020, with projections exceeding 16% by 2030. This phenomenon is further compounded by economic vulnerability, as only 23% receive a pension, 5% actively contribute to the pension system, and 70% make no contributions, which, in turn, increases the risk of institutionalization and abandonment^12^.

National studies demonstrate that social support and functional capacity are determinants of quality of life among older adults^13^, which should be understood in conjunction with environmental and cultural aspects, access to services, personal satisfaction, and recreational activities^14^. In this context, caregivers require evidence-based training to strengthen the physical and emotional well-being of older adults^15^, while care planning should incorporate strategies to promote physical and cognitive functioning and social participation^16^.

From an interdisciplinary approach, nursing holds a strategic position in the comprehensive assessment and early detection of risks among institutionalized individuals^17^. In addition to providing direct care, professionals should influence public policies aimed at reducing inequalities, ensuring access to services, and promoting social inclusion^18^.

The FUMAT scale has become the standard tool for assessing quality of life in nursing homes. Based on the model developed by Schalock and Verdugo, the scale assesses eight fundamental dimensions: emotional well-being, physical well-being, material well-being, self-determination, personal development, interpersonal relationships, social inclusion, and rights^6^. Its excellent psychometric properties have enabled its use in numerous studies, including one conducted in Cúcuta, Colombia, aimed at characterizing quality-of-life profiles^19^. The scale facilitates the planning of personalized care, comprehensive monitoring of physical, psychological, and social well-being, and identification of areas of vulnerability^20^–^23^. Nevertheless, the necessity persists to generate contextualized evidence that reflects the sociocultural realities of institutional settings. In response to this, the present study aimed to analyze the factors associated with the quality-of-life profiles among institutionalized older adults, contributing to the design of comprehensive, ethical, and humanized care strategies.

## Materials and Methods

**Design**

A cross-sectional analytical study was conducted with institutionalized older adults in a nursing home in northern Bucaramanga during 2024. The population under study consisted of users of the Albeiro Vargas and Ángeles Custodios Foundation, which serves approximately 300 individuals from a low socioeconomic status. The sample size (n = 250) was determined using Epidat 3.1, taking as a reference the variability in physical well-being reported in a Spanish study (mean 44.52 ± 22.55)^24^, with an alpha error of 0.03 and a beta error of 0.80. Furthermore, the findings of Morales et al. in Ecuador^25^, which reported a 62.04% prevalence of excellent quality of life, were taken into consideration. This resulted in an alpha error of 0.024 and a beta of 0.80.

Convenience sampling was used. Individuals aged 60 years or older were included if they were permanent residents for at least three months and had a reliable informant. Individuals with terminal illness, acute psychiatric disorders, or clinical instability were excluded from the study.

Data was collected by the principal investigator, a nurse in postdoctoral training, following a three- month period of interaction and observation at the nursing home, which enabled an understanding of the participants’ social and functional dynamics. The FUMAT scale was administered with the support of gerontologists and caregivers, who acted as key informants to ensure the validity and reliability of the responses. The remaining instruments were administered directly by the researcher in accordance with standardized procedures. This methodological strategy ensured objectivity, consistency, and accuracy, faithfully reflecting the real conditions of institutionalized older adults.

**Instruments**

The FUMAT scale is an instrument designed to evaluate the quality of life of older adults, including those with severe physical disability or cognitive impairment, based on the model developed by Schalock and Verdugo^4^. It exhibits high content validity and internal consistency (α=0.95) and adequate reliability (r=0.90). Its application, intended for professionals familiar with the individual, does not require the direct participation of the person assessed and is based on observable behaviors. The scale consists of 57 items phrased in the third person, with four response options, and assesses eight dimensions: emotional well-being, interpersonal relationships, material well-being, personal development, physical well-being, self-determination, social inclusion, and rights^6^,^26^. The scale enables the creation of an individual profile using standardized scores that are comparable across dimensions, as well as an overall index and a percentile that reflect the level of quality of life, with higher values indicating better well-being.

Additionally, functional assessment instruments widely used in nursing practice were employed. The Barthel Index is a widely used instrument for assessing independence in basic activities of daily living among individuals with functional impairment, especially older adults. The instrument has demonstrated high reliability and validity across multiple studies, with internal consistency exceeding 0.85. The Barthel Index assesses ten basic activities and assigns scores ranging from 0 to 100, with lower scores denoting increased dependence: 0 to 20 (total dependence), 21 to 60 (severe dependence), 61 to 90 (moderate dependence), 91 to 99 (mild dependence), and 100 (independence). This scale enables the identification of the level of functional independence, guides care planning, and supports the assessment of the effectiveness of interventions aimed at maintaining or restoring autonomy^27^–^29^.

The PULSES profile assesses disability and functional status across different clinical settings, including rehabilitation and older adult care. It examines six dimensions: physical condition, upper and lower limb function, sensory stability, excretory function, and social and mental status. The instrument has demonstrated high interobserver reliability (r > 0.80) and good internal consistency (α = 0.75– 0.90). Each dimension is scored on a scale from 1 (independence) to 4 (severe dependence), with a total possible score of 6 to 24 points. Lower values indicate higher functional independence. Its comprehensive score facilitates categorization of disability, guides clinical decisions, prioritizes interventions, and monitors functional progression, making it a widely used instrument for planning autonomy-centered care^30^–^32^.

The FRAIL scale identifies frailty in older adults and enables the prediction of risks such as disability, hospitalization, and mortality. It assesses five dimensions: fatigue, resistance, aerobic capacity, illness, and loss of weight. Each item is scored 0 or 1, yielding a total score ranging from 0 to 5 that classifies individuals as robust (0), pre-frail (1–2), or frail (3–5). The scale has been validated across various geriatric populations and clinical settings, demonstrating high sensitivity, specificity, and interobserver reproducibility^33^–^35^.

The Downton Fall Risk Index assesses the risk of falls in older adults through five items: previous falls, medication use, sensory deficits, mental state, and gait. Each item is scored as 0 or 1, with a maximum total score of 5; values of 3 or higher indicate high risk. The instrument has demonstrated good sensitivity and specificity in detecting the risk of falls in the geriatric population; however, its clinical application has been characterized by high sensitivity and low specificity^36^–^38^.

The Pfeiffer’s SPMSQ is a brief instrument for detecting cognitive impairment in older adults. It assesses orientation, memory, calculation, and general knowledge through ten items administered rapidly. The instrument demonstrated good interobserver reliability (0.73) and intraobserver reliability (0.93), supporting its consistency. Each incorrect response is counted as an error, and the total number of errors is used to classify cognitive impairment as follows: 0–2 errors (normal), 3–4 (mild), 5–7 (moderate), and 8–10 (severe)^39^–^41^.

**Data analysis**

A descriptive analysis was performed according to the nature of the variables. Absolute and relative frequencies were calculated for qualitative variables, while medians and interquartile ranges were reported for quantitative variables. Distributional assumptions were assessed using the Shapiro– Francia test. Box-and-whisker plots were generated to illustrate the percentiles of the FUMAT Quality of Life Index (FUMAT-QOLI) dimensions.

The outcome variable, the FUMAT Quality of Life Index, was categorized into tertiles; the first and second tertiles were grouped and compared with the third tertile, resulting in the creation of a dichotomous nominal variable. Pearson’s chi-square test or Fisher’s exact test was used for qualitative variables, and the Mann–Whitney U test was applied for quantitative variables.

Correlation analysis was conducted using Spearman’s rho, and a correlation matrix was generated to examine the relationships between age and the scores of the FUMAT-QOLI, the Barthel Index, the PULSES profile, the FRAIL scale, the Downton Fall Risk Index, and the SPMSQ. To identify factors associated with the upper tertile of the QLI, multivariate logistic regression was performed following the steps proposed by Hosmer and Lemeshow^42^, including variables with p <0.05 in the bivariate analysis and age, given its clinical relevance. Model’s fit was assessed using the Hosmer–Lemeshow test. All analyses were conducted using Stata v17 and, in accordance with the open science guidelines, the data were deposited in Mendeley Data^43^.

**Ethical considerations**

The research was conducted in accordance with the principles of confidentiality and data protection, thereby ensuring that participants were the focus of ethical protection. Data collection was conducted with only free, voluntary, informed consent, and all information was stored in protected files under the custody of the principal investigator, thereby preserving participants’ privacy and confidentiality. Data obtained through instruments, techniques, and interviews were used exclusively for the purposes of the study. The nursing home provided adequate spaces that ensured privacy, confidentiality, and compliance with biosafety standards. Participants’ rights were safeguarded in accordance with national and international regulations, ensuring that neither healthcare staff nor the services provided were affected during the research process. The study received approval from the Bioethics Committee, as documented in Minutes No. 007 dated April 17, 2023.

## Results

Of the 250 participants, 63.60% (n=159) were female, and 36.40% (n=91) were male, with a median age of 77.6 years (interquartile range [IQR: 70.23–84]). Table 1 presents a comparison between participants in the initial two tertiles of the FUMAT Quality of Life Index (FUMAT-QOLI) and those in the third tertile (highest scores). Statistically significant age differences were identified: participants in terciles 1 and 2 had a median age of 79.84 years (IQR: 72.38–84.95), whereas those in the third tertile had a median age of 73.65 years (IQR: 67.86–79.23) (p < 0.001), indicating a better quality of life among the younger age groups.

Regarding educational level, participants with no education or only primary education were mainly concentrated in the third tertile, whereas those with secondary or professional education were more frequently represented in tertiles 1 and 2. This suggests an association between educational attainment and self-perceived well-being.

Regarding personal medical history, statistically significant associations (p <0.05) were observed among participants with psychiatric, degenerative, and respiratory diseases, as well as among those who reported the use of tranquilizers or antidepressants. Similarly, individuals with mental, cognitive, motor, or multiple disabilities exhibited a lower proportion among those with the highest FUMAT- QOLI scores. Conversely, individuals who reported engaging in regular physical activity and productive activities were concentrated in the third tertile, supporting the positive relationship between active aging and the perception of a better quality of life.

**Table 1:** Comparison of FUMAT-QOLI tertiles with participant characteristics

Characteristics	Total % (n) (250)	Tertiles 1/2 lower level 7–76 (171)	Tertile 3 higher level 77–93 (79)	p-value
Sex				0.573
Female	63.60 (159)	64.91 (111)	60.76 (48)	
Male	36.40 (91)	35.09 (60)	39.24 (31)	
Age, median (IQR)	77.60 [70.23; 84.00]	79.84 [72.38; 84.95]	73.65 [67.86; 79.23]	<0.001
Marital status				0.852
Married/Cohabiting	25.15 (43)	25.15 (43)	24.05 (19)	
Single/Divorced/Widowed	74.85 (128)	74.85 (128)	75.95 (60)	
Level of education				0.002
None	25.60 (64)	22.22 (38)	32.91 (26)	
Primary education	55.20 (138)	52.63 (90)	60.76 (48)	
Secondary education	8.80 (22)	11.11 (19)	3.80 (3)	
Professional education	3.60 (9)	4.09 (7)	2.53 (2)	
Do not know / No response	6.80 (17)	9.94 (17)	0	
Health insurance regime				0.704
Subsidized	80.80 (202)	80.70 (138)	81.01 (64)	
Contributory	17.60 (44)	18.13 (31)	16.46 (13)	
Other	1.60 (4)	1.17 (2)	2.53 (2)	
Personal medical history				
Cardiovascular	63.60 (159)	63.74 (109)	63.29 (50)	1.000
Metabolic	28.00 (70)	25.15 (43)	34.18 (27)	0.172
Psychiatric	38.40 (96)	47.37 (81)	18.99 (15)	<0.001
Degenerative	35.20 (88)	42.11 (72)	20.25 (16)	0.001
Respiratory	16.40 (41)	21.05 (36)	6.33 (5)	0.003
Neoplasia-cancer	4.80 (12)	6.43 (11)	1.27 (1)	0.110
Medications				
Diuretics	19.20 (48)	19.88 (34)	17.72 (14)	0.687
Tranquilizers	32.80 (82)	42.11 (72)	12.66 (10)	<0.001
Antiparkinsonian	6.00 (15)	7.02 (12)	3.80 (3)	0.401
Antidepressants	27.60 (69)	36.26 (62)	8.86 (7)	<0.001
Antihypertensive	56.40 (141)	55.56 (95)	58.23 (46)	0.692
Impairment				
Vision impairment	54.00 (135)	54.97 (94)	51.90 (41)	0.650
Hearing loss	27.20 (68)	29.82 (51)	21.52 (17)	0.170
Mental	34.80 (87)	45.03 (77)	12.66 (10)	<0.001
Cognitive	35.20 (88)	46.78 (80)	10.13 (8)	<0.001
Motor	36.40 (91)	46.20 (79)	15.19 (12)	<0.001
Multiple	39.20 (98)	52.05 (89)	11.39 (9)	<0.001
Physical activity	52.80 (132)	43.86 (75)	72.15 (57)	<0.001
Productive activity	54.00 (135)	43.86 (75)	75.95 (60)	<0.001

P-values: Pearson’s chi-square test and Fisher’s exact test for qualitative variables. Mann-Whitney U test for age.

**Figure 1:**
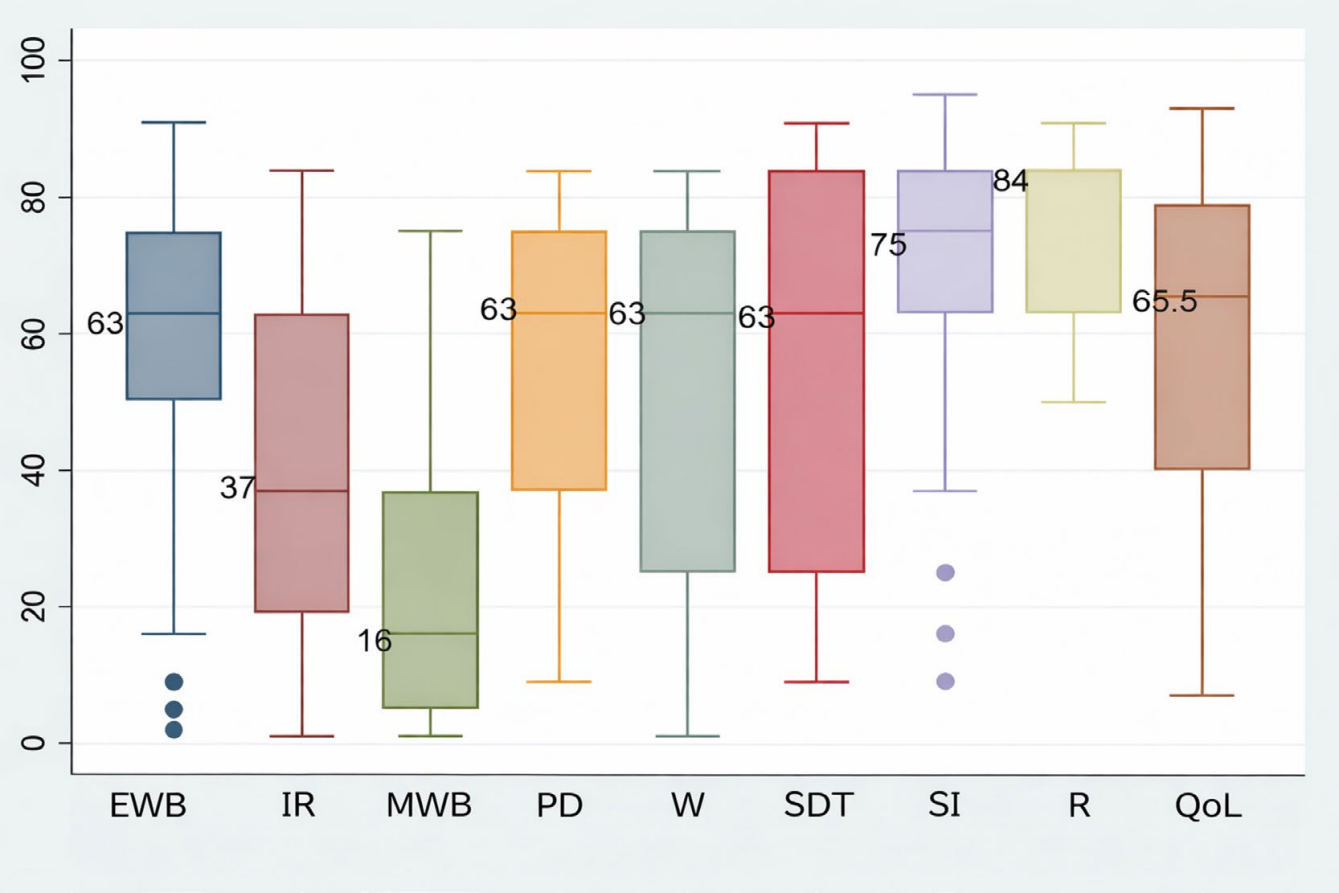
Percentiles of dimensions and the FUMAT quality of life index

The medians of the percentiles for the FUMAT-QOLI dimension are shown in Figure 1. The dimensions with the lowest scores were material well-being, with a median of 16 (IQR: 5–37), followed by interpersonal relationships, with a median of 37 (IQR: 19–63). Conversely, the highest scores were observed for the rights dimension, with a median of 84 (IQR: 63–84), and social inclusion, with a median 75 (IQR: 63–84). Overall, the FUMAT-QOLI showed a median score of 65.5 (IQR: 40–79).

*EWB: Emotional well-being, IR: Interpersonal relationships, MWB: Material well-being, PD: Personal development, W: Physical wellbeing, SDT: Self-Determination, SI: Social inclusion, R: Rights, QoL: Quality of life Index.*

**Figure 2:**
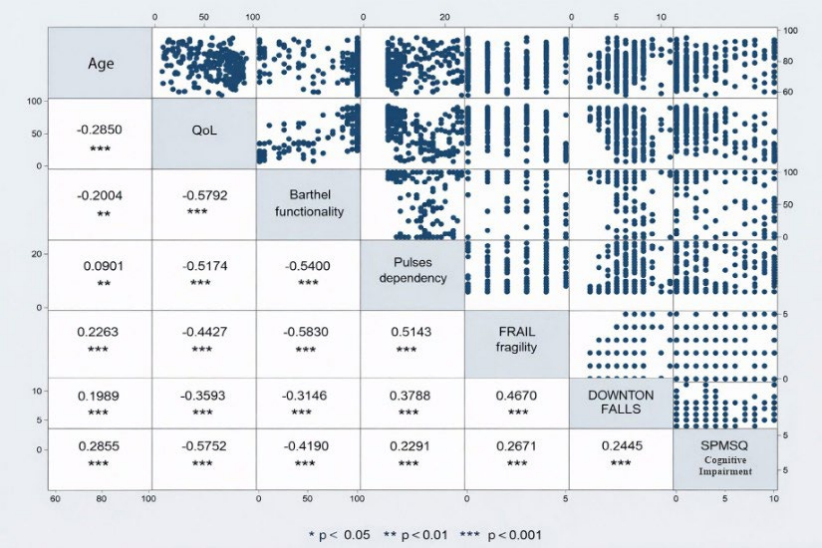
Correlation matrix of variables

In Spearman’s correlation matrix (Figure 2), age exhibited a negative and statistically significant correlation with the FUMAT-QOLI (Rho = –0.29, p < 0.001) and with functional independence, as measured by the Barthel Index (Rho = –0.20, p <0.01). Although both associations were statistically significant, their magnitudes corresponded to weak correlations; therefore, while increasing age tended to be associated with reduced functional independence and quality of life, this relationship was not strong. No statistically significant correlation was identified between age and the PULSES profile of dependence.

Spearman’s rho. p-value: * <0.05 ** <0.01 *** <0.001

The strongest correlations were observed between the FUMAT-QOLI and the functional status (Barthel Index) (Rho = 0.5792, p < 0.001), suggesting a direct association between functional autonomy and a better perceived well-being. Similarly, a strong negative correlation was found between FUMAT-QOLI and cognitive impairment (SPMSQ) (Rho = -0.5752, p < 0.001), indicating that cognitive impairment is associated with lower quality of life.

Likewise, significant associations were observed among frailty (FRAIL scale), dependence (PULSES profile), and the risk of falls (Downton Index), with positive correlation coefficients among these measures, indicating their interdependence in the overall functional status of institutionalized older adults.

Table 2 presents the comparison between tertiles of the FUMAT-QOLI and the categories of functional status, dependence, frailty, risk of falls, and cognitive impairment. The results indicate statistically significant associations between the quality-of-life index and the Barthel Index, the PULSES profile, the FRAIL scale, and the SPMSQ (p < 0.001), whereas no statistically significant differences were observed for the Downton Fall Risk Index (p = 0.384).

**Table 2:** Comparison of FUMAT-QOLI tertiles with functional status, dependence, frailty, risk of falls, and cognitive impairment.

Characteristics	Total % (n) (250)	Percentile FUMAT-QOLI		p-value
		Tertile 1/2 7–76 (171)	Tertile 3 77–93 (79)	
Functional status (Barthel Index)				<0.001
Total dependence	9.60 (24)	14.04 (24)	0	
Severe dependence	4.80 (12)	7.02 (12)	0	
Moderate dependence	6.40 (16)	9.36 (16)	0	
Mild dependence	14.00 (35)	15.20 (26)	11.39 (9)	
Independent	65.20 (163)	54.39 (93)	88.61 (70)	
Dependence (PULSES profile)				<0.001
Mild impairment	49.20 (123)	36.84 (63)	75.95 (60)	
Moderate impairment or dependence	15.20 (38)	16.37 (28)	12.66 (10)	
Severe impairment or dependence	35.60 (89)	46.78 (80)	11.39 (9)	
Frailty (FRAIL scale)				<0.001
Not frail	76.80 (192)	68.42 (117)	94.94 (75)	
Frail	23.20 (58)	31.58 (54)	5.06 (4)	
Downton Fall Risk				0.384
Moderate risk	2.40 (6)	1.75 (3)	3.80 (3)	
High risk	97.60 (244)	98.25 (168)	96.20 (76)	
Cognitive impairment (SPMSQ)				<0.001
Normal	60.00 (150)	46.20 (79)	89.87 (71)	
Minimal	6.00 (15)	8.19 (14)	1.27 (1)	
Moderate	17.20 (43)	21.64 (37)	7.59 (6)	
Severe	16.80 (42)	23.98 (41)	1.27 (1)	

p-value: Pearson’s chi-square test and Fisher’s exact test

Regarding functional status, as measured by the Barthel Index, the categories of total, severe, and moderate dependence were observed exclusively among participants in tertiles 1 and 2. Conversely, no participants in the third tertile (indicating better quality of life) presented these conditions. In this latter group, 88.61% were classified as independent, compared with 54.39% in the lower tertiles.

With respect to functional dependence (PULSES profile), the mild impairment category predominated in the third tertile (75.95%), whereas severe dependence was more prevalent in tertiles 1 and 2 (46.78%), confirming an inverse relationship between dependence and quality of life.

According to the FRAIL scale, 94.94% of participants in the third tertile were classified as not frail, compared with 68.42% in tertiles 1 and 2. This finding indicates a significant association between lower frailty and better quality of life.

Although no statistically significant differences were observed in the Downton fall risk index, a homogeneous high-risk tendency was maintained across all groups.

Finally, according to Pfeiffer’s SPMSQ, 89.87% of participants in the third tertile were classified as having intact cognitive function, whereas only 46.20% of those in the lower tertiles fell into this category. Moderate and severe impairment levels were concentrated in tertiles 1 and 2, with proportions of 21.64% and 23.98%, respectively, reinforcing the influence of cognitive status on the overall perception of quality of life.

Multivariate analysis showed that participants with cognitive impairment were less likely to be in the highest tertile of the FUMAT-QOLI scores (OR = 0.34, 95% CI: 0.12; 0.91, p = 0.033), as well as participants with motor impairment (OR = 0.30, 95% CI: 0.14; 0.64, p = 0.002). When participants reported engaging in physical activity and productive activities, they showed a greater likelihood of belonging to the group with the highest scores. While these associations were not statistically significant, a trend was identified. See Table 3.

**Table 3:** Multivariate logistic regression model for belonging to the highest tertile of FUMAT- QOLI scores

Variables	OR (95% CI)	p-value
Age	0.98 (0.94 - 1.01)	0.345
Respiratory condition	0.39 (0.13 - 1.18)	0.099
Impairment		
Mental	0.48 (0.19 - 1.21)	0.121
Cognitive	0.34 (0.12 - 0.91)	0.033
Motor	0.30 (0.14 - 0.64)	0.002
Physical activity	1.70 (0.87 - 3.29)	0.116
Productive activity	2.01 (0.98 - 3.86)	0.054

p-value: Wald test.

## Discussion

The findings of this study have significant implications for nursing practice in the field of gerontology. The association between engagement in productive and physical activities and higher scores on the FUMAT scale underscores the importance of nursing interventions that promote mobility, adapted exercise, and social integration as essential strategies for preserving and enhancing quality of life in older adults. Likewise, identifying cognitive and motor disabilities as factors that reduce quality of life underscores the need for a preventive and early care approach, in which nursing assumes an active role in detecting functional impairments and designing personalized care plans.

The study identified that participation in productive and physical activities is strongly associated with higher scores on the FUMAT scale. Similar results were reported in a study conducted in Chile among older women, which observed significant differences in subjective quality-of-life perceptions between physically active and inactive individuals, with the former reporting higher levels of satisfaction and overall well-being following the COVID-19 pandemic^44^.

Compared with international studies that have employed the FUMAT scale, the dimension of material well-being exhibited the lowest scores in this study. This finding aligns with regional evidence from Paraguay^45^, where a study among 33 residents of peri-urban nursing homes in Asunción reported material well-being as one of the lowest-scoring dimensions (45 ± 30). These figures underscore the role of social determinants in quality of life. Studies conducted in Ecuador^22^, Cúcuta, Colombia^46^, and Mexico^47^ reported analogous trends, namely, lower ratings of material well-being compared with other dimensions. By contrast, a study conducted in Spain that employed a short version of the FUMAT scale (24 items) identified social inclusion, self-determination, and physical well-being as the lowest-scoring dimensions. These findings suggest that differences in sociocultural context and in the version of the instrument may modulate the dimensional profiles observed^48^.

In this study, the dimensions that obtained the highest scores were rights, social inclusion, and self-determination, reflecting a positive perception of aspects related to participation, integration, and personal autonomy. Similar results were observed in a study conducted in Ecuador^22^ involving 137 older adults, in which the highest-scoring dimensions were self-determination (63 points) and personal development and rights (50 points each). Similarly, a study conducted in Spain^24^ with a sample of 25 older adults revealed that the highest-scoring dimensions were personal development (57 points), social inclusion (52 points), and physical well-being (45 points). These findings confirmed the consistency of these domains as relevant indicators of perceived well-being in old age.

These results enable the identification of the material well-being dimension of the scale as the lowest-scoring across the different studies, indicating that it remains a factor limiting the quality of life of institutionalized older adults. This finding underscores the importance of strengthening public policies^49^ to protect the economic well-being of this population.

The present study demonstrated that cognitive and motor impairments significantly impact quality of life, consistent with previous research conducted in similar contexts, in which frailty and cognitive impairment have been identified as determinants of perceived well-being among older adults^50^–^53^. These findings enrich the body of knowledge in gerontological nursing by providing local evidence that supports person-centered care practices and encourages professionals to strengthen comprehensive assessment as a basis for effective interventions. Promoting physical activity, preventing cognitive and motor impairments, and ensuring social inclusion not only enhance health outcomes but also reflect nursing’s commitment to the humanization of care for institutionalized older adults.

Another relevant point of comparison emerges from the analysis of the QOLI in relation to mental health variables. In the present study, psychiatric history and the use of tranquilizers or antidepressants were found to be associated with lower quality of life. This finding is consistent with other studies highlighting the negative impact of mental disorders on older adults^54^. However, the present study underscores the need for nursing interventions aimed at promoting mental health and reducing polypharmacy. These interventions have the potential to enhance the overall well-being of this population^55^.

Among its strengths, the study provides robust empirical evidence through a solid methodological design based on validated instruments and multivariate analysis. As a result, factors associated with quality of life were identified with high reliability. Furthermore, the research encompasses a substantial sample of older adults, thereby enhancing its external validity and enabling the extrapolation of its findings to similar contexts.

With regard to contributions to nursing practice, education, and research, the study highlights key elements for strengthening care, such as the need to include person-centered interventions that promote physical activity and social participation as effective strategies for improving quality of life in nursing homes^56^. Furthermore, the role of nursing in assessing physical and cognitive functioning is essential for early detection of impairments that affect quality of life^57^.

From the perspective of nursing education, new directions emerge for undergraduate and graduate nursing curricula, including the comprehensive assessment of older adults, the use of validated scales in this population, such as the FUMAT scale, and care planning focused on the dimensions of material, emotional, and social well-being^26^. In this way, nursing professional competence can be strengthened, promoting active aging among individuals both within and without institutionalization.

Amongthe limitations tobe considered, first, the study was conducted in a single nursing home, limiting the generalizability of the results to other older adult populations with different sociodemographic characteristics or living conditions.

Regarding future lines of research, it would be relevant to conduct longitudinal studies to assess changes in quality of life in this population over time and to identify more accurate predictors. Examples include a study conducted in Spain that examined the effects of a community-based home care intervention^58^; research in Peru reporting the impact of a social program among older adults living in extreme poverty, with the participation of 634 older adults^59^; and studies assessing outcomes in nursing homes involving individuals with cognitive impairment^56^. It is also recommended to expand the research to different types of older people’s homes, including urban and rural settings^60^, to evaluate potential variations in quality of life according to context, including hospital settings^61^.

In view of the above, new lines of research may be considered to evaluate the effects of nursing interventions on the prevention or limitation of disability and cognitive impairment. In this way, best care practices can be explored that foster the inclusion and social participation of older adults across different clinical and community settings, including nursing homes.

Furthermore, it would be relevant to consider validating the 24-item FUMAT scale^62^. Measuring quality of life in individuals with intellectual disabilities or multiple disabilities^63^ also remains a challenge, as does examining the effects of various interventions, such as physical activity plans, dance programs, music therapy^64^–^67^, and the prevention of polypharmacy^68^, among others that involve the role of gerontological nursing. These efforts would contribute to continued progress toward excellence in care, promoting humanized care, active aging, and dignified treatment of older adults.

## Conclusions

This study highlights the positive influence of participation in productive activities and physical activity on the quality of life of older adults and encourages health professionals to prioritize the prevention of cognitive and motor impairments in this population. Furthermore, this study makes a significant contribution to the strengthening of gerontological practice by identifying key factors that impact the quality of life of institutionalized older adults. 
